# Comparison of Aquitaine and Rioja Red Wines: Characterization of Their Phenolic Composition and Evolution from 2000 to 2013

**DOI:** 10.3390/molecules22020192

**Published:** 2017-01-24

**Authors:** Cindy Quaglieri, Noelia Prieto-Perea, Luis Angel Berrueta, Blanca Gallo, Zurine Rasines-Perea, Michael Jourdes, Pierre-Louis Teissedre

**Affiliations:** 1 University Bordeaux, ISVV, EA 4577 Œnologie, F-33140 Villenave d’Ornon, France; cindyquaglieri@gmail.com (C.Q.); zuri.rasines@gmail.com (Z.R.-P.); michael.jourdes@u-bordeaux.fr (M.J.); 2 INRA, ISVV, USC 1366 Œnologie, F-33140 Villenave d’Ornon, France; 3 Departamento de Química Analítica, Facultad de Ciencia y Tecnología, Universidad del País Vasco/Euskal Herriko Unibertsitatea (UPV/EHU), P.O. Box 644, 48080 Bilbao, Spain; noeliap_89@hotmail.com (N.P.-P.); luisangel.berrueta@ehu.eus (L.A.B.); blanca.gallo@ehu.eus (B.G.)

**Keywords:** wine composition, pigments, tannins, anthocyanins, pyranoanthocyanins, HPLC-DAD-ESI-MS/MS, component levels, Rioja, Aquitaine

## Abstract

Wine chemical analysis was carried out on 194 commercial blended red wines produced by two major wine-growing areas—the Aquitaine (France) and Rioja (Spain) regions—in order to compare the wines of both regions. Anthocyanins and derived pigments, tannins and derivatives were identified and quantified by HPLC-DAD-ESI-MS/MS (high pressure liquid chromatography coupled to diode array detector and mass spectrometry using the electrospray ionization interface). Mean degree of polymerization (mDP) was determined. The influence of the wine-growing region and the predominance of the properties of some grape varieties used are confirmed by the significant differences observed between both regions. Rioja and Bordeaux “generic” (Bordeaux and Bordeaux-Supérieur appellations) red wines showed the highest anthocyanic content and the highest mDP, as these wines are in a majority made from Merlot (Bordeaux “generic”) and Tempranillo (Rioja). On the contrary, Bordeaux “specific” regions (Blayais, Médoc, Graves, and Libournais) showed the red wines with the highest total phenolic content and tannin concentration, as the predominant grape variety used is Cabernet Sauvignon. A principal component analysis (PCA) and a hierarchical ascendant classification (HAC) suggesting patterns between the chemical parameters and the distribution of the red wines in three groups were proposed. The comparison of the two wine-growing areas also reveals some similarities between the various grape varieties used. A general effect of a progressive decrease in anthocyanins, anthocyanin-derived pigment and tannins is observed for older wines.

## 1. Introduction

Phenolic composition, especially anthocyanin and tannin content, is one of the most important wine attributes. On the one hand, the red wine colour is due to the presence of anthocyanins, directly extracted from grape skins, and anthocyanin-derived pigments, formed during wine-making and ageing. Even though anthocyanins are the principal source of colour for young red wines, they are very reactive at wine pH. A progressive diminution in these monomeric anthocyanins and a simultaneous increase of polymeric pigments as well as color intensity and stability are observed. This is the result of interactions among other phenolic compounds, also extracted during the wine-making process [1,2], leading to the stabilization of wine colour. They can react with each other to form anthocyanin oligomers [3,4], or with flavan-3-ol units through a direct condensation or the formation of an ethylidene bridge, leading to the formation of stable red-purple pigments [5]. Anthocyanins also undergo a cycloaddition mechanism to form pyranoanthocyanins, which are distributed in different groups depending on the nature of the structure reacting with the anthocyanin: pyruvic acid (A-type vitisins) [6,7,8], acetaldehyde (B-type vitisins) [9,10], acetoacetic acid (methylpyrano-anthocyanins) [11,12], vinylphenols (vinylphenol pyranoanthocyanins) or vinylflavanols (flavanol pyranoanthocyanins) [13,14], 4-vinylguaiacol [15], glyoxylic acid [16] or α-ketoglutaric acid [9]. Pyranoanthocyanins are more stable than flavan-3-ol/anthocyanin adducts as their additional pyranic ring formed during the cycloaddition step provides more stability towards nucleophilic attack [17].

On the other hand, flavan-3-ols, such as procyanidins (also called condensed tannins), are responsible for wine astringency and bitterness [18,19]. During ageing, tannin polymerization is associated to more softened tannins during wine-tasting [20]. These are also involved in the formation of polymeric anthocyanin/flavan-3-ol or flavan-3-ol/flavan-3-ol adducts, by the way of a direct condensation or indirect condensation through the formation of an ethyl bridge [5].

Previous studies have characterized phenolic contents of one [21,22,23] or several varietal red wines [24,25], but little is known about the phenolic composition of red wines assembled with two or more grape varieties, from different wine-growing regions compared together in the same study.

This study aims to investigate the phenolic composition of red wines from two major wine-growing areas: Aquitaine in the Southwest of France (44°85′ north and 2°44′ west), and Rioja in the North of Spain (42°20′ north and 2° west). The Aquitaine region has an oceanic climate, whereas the Rioja region has a continental climate. Both are among the most important wine producing regions in the World. Whereas the main red grape varieties used by Rioja wine producers are Tempranillo, Grenache and Graciano, the major red cultivars in Aquitaine are Cabernet Sauvignon, Cabernet Franc and Merlot. The Rioja region is divided in three subregions: Rioja Alta, Rioja Baja and Rioja Alavesa. The Aquitaine region is divided in several subregions, with Médoc, Graves, Blayais and Libournais being some of the most important. Generic Bordeaux denominations (Bordeaux and Bordeaux superior) can be produced in all Aquitaine regions.

Sampling was performed in order to provide a representative overview of the market in each region with a vertical distribution using the red wine samples from 2000 to 2013 vintages (randomly gathered samples). These samples are blended wines, that have been collected and analyzed in a short period of time (February–June 2014), that have been produced using different grape varieties depending on the region and subregion and showing various wine-ageing states and conditions. The investigation of their anthocyanin and flavan-3-ol compositions, anthocyanin-derived pigments contents and mean degree of polymerization aims to highlight a distribution of the samples according to the wine-growing areas, proposing causes such as variety and vintage for any found differences.

## 2. Results

In our study, the phenolic composition of 194 different blended red wines made at different times and distributed between two major wine-growing regions (Aquitaine and Rioja) was analyzed. For the Aquitaine region, wines are divided into six categories, wines come from four subregions: Blayais & Bourgeais, Médoc, Graves, Libournais on the one hand, which represent “Bordeaux specific” appellations, and Bordeaux and Bordeaux-Supérieur on the other hand, characterizing “Bordeaux generic” appellations. The Rioja region is divided into three subregions: Rioja Alta, Rioja Baja and Rioja Alavesa. The results are presented according to three main contents: anthocyanin and derived-pigment contents, tannin contents and mean degree of polymerization (mDP).

### 2.1. Descriptive Analysis of the Phenolic Characterization of Wine Samples: Distribution by Wine-Growing Areas and Vintages

#### 2.1.1. Anthocyanins and Derived Pigments Contents

**Figure A1:**
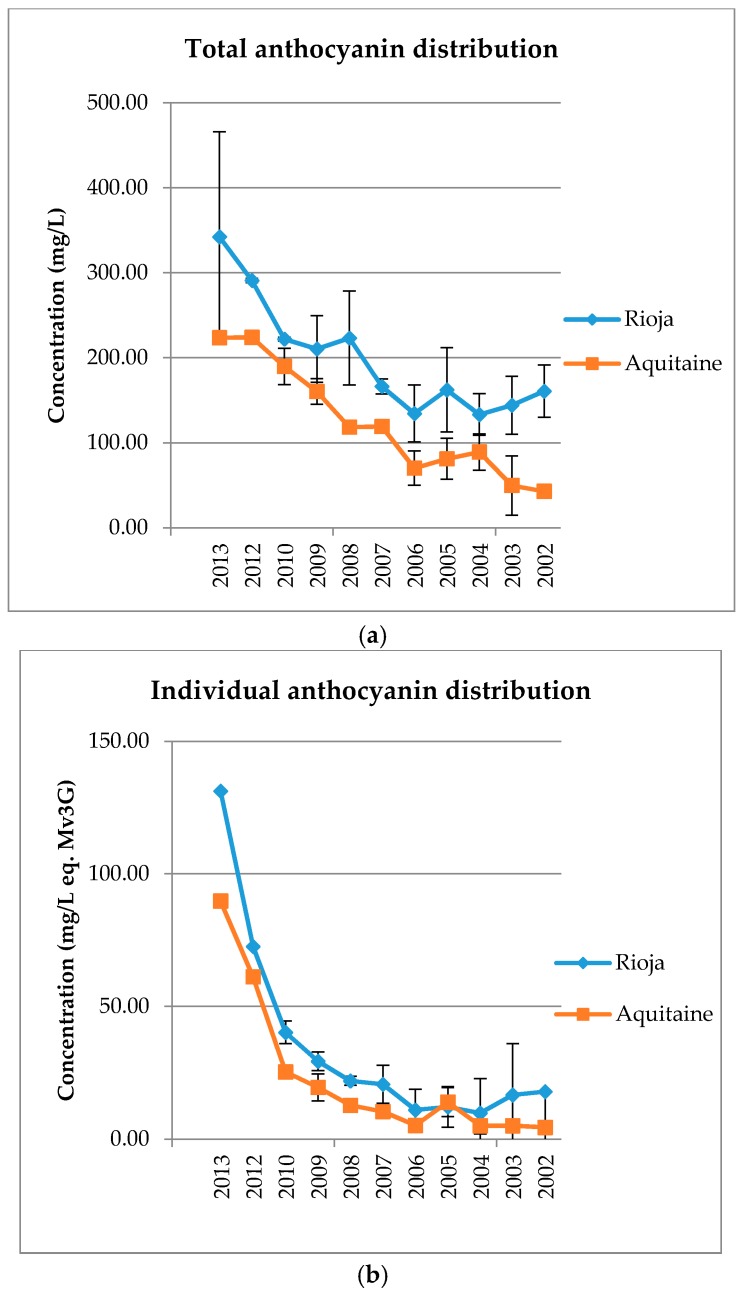
Total (**a**) and individual (**b**) anthocyanin distribution for Rioja and Aquitaine wines. Error bars are standard deviation of wine samples within each category.

The anthocyanin content distributions for each wine by growing area and by vintage (average values of the data set) are presented in Table 1 and Table 2. The distributions of individual anthocyanins, pyranoanthocyanins and polymerized pigments are also evaluated (Table 1A). Total anthocyanin contents are lower in older wines than in the younger wine samples. Separately, the same tendency is observed for Rioja and Aquitaine wines, but with different concentration levels (Figure A1). From 2002 to 2013, Rioja red wines show higher concentrations of total and individual anthocyanins (means of +109% and +93% towards Aquitaine wines, respectively). “Bordeaux specific” appellations (Blayais, Libournais, Médoc and Graves) show lower concentrations of individual anthocyanins, with totals of 8.10, 14.62, 18.63 and 25.61 mg/L, in eq. malvidin-3-*O*-glucoside (Mv3G), respectively, than “Bordeaux generic” and the Rioja regions (Alta, Alavesa, “Bordeaux generic” and Baja with totals of 31.40, 46.03, 47.19 and 58.92 mg/L in eq. Mv3G, respectively). Furthermore, Rioja wines show higher concentrations of coumaroylated pigments and lower levels of acetylated ones than Bordeaux wines, as a consequence of differences in the major grape varieties used in each region, as below discussed.

Total and individual anthocyanin contents show a joint evolution with a correlated decrease of each attribute for older wines as compared to younger ones (Figure A1). Separately, the same tendency is observed for Rioja and Aquitaine wines, but with different concentration levels (Table 1A). For Rioja wines, the levels of total and individual anthocyanins are well correlated to the wine “type” and hence to barrel ageing time: Gran Reserva wines show the lowest concentrations (means of 177.64 mg/L and 13.43 mg/L for total and individual anthocyanins, respectively), and Joven the highest (means of 299.98 mg/L and 88.39 mg/L). The decreasing concentrations of anthocyanins are due to chemical reactions involving these molecules to form anthocyanin-derived adducts that enhance wine colour and hue.

**Figure 1:**
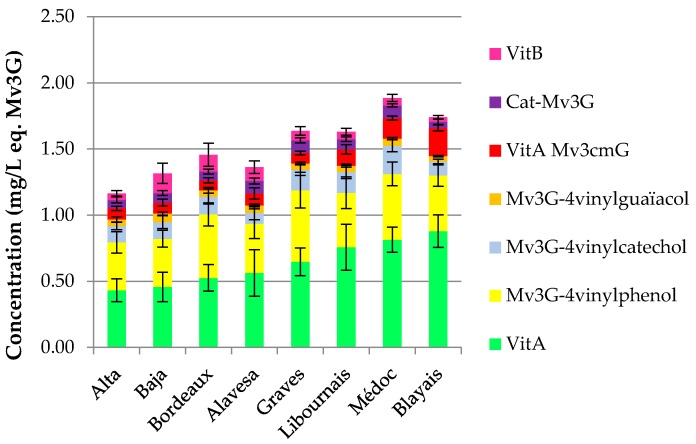
Pyranoanthocyanins and polymerized pigments for each wine-growing region. VitB*,* vitisin B; Cat-Mv3G, direct catechin-malvidin-3-*O*-glucoside adduct; VitA Mv3cmG, vitisin A-malvidin-3-*O*-*p*-coumaroylglucoside; Mv3G-4-vinylguaiacol, pyranomalvidin-3-*O*-glucoside-4-vinylguaiacol; Mv3G-4-vinylcatechol, pyranomalvidin-3-*O*-glucoside-4-vinylcatechol; Mv3G-4-vinylphenol, pyranomalvidin-3-*O*-glucoside-4-vinylphenol; VitA, vitisin A. Error bars are standard deviation of wine samples within each category.

The distribution of pyranoanthocyanins and polymerized pigments for wines from each area is shown in Figure 1. On the one hand, “Bordeaux specific” wines show a higher content in vitisin A (Mv3G-pyruvic acid) than Rioja and “Bordeaux generic” wines, distributing the wine samples according to their origin and hence varieties, as initially shown for total and individual anthocyanin contents.

**Figure 2:**
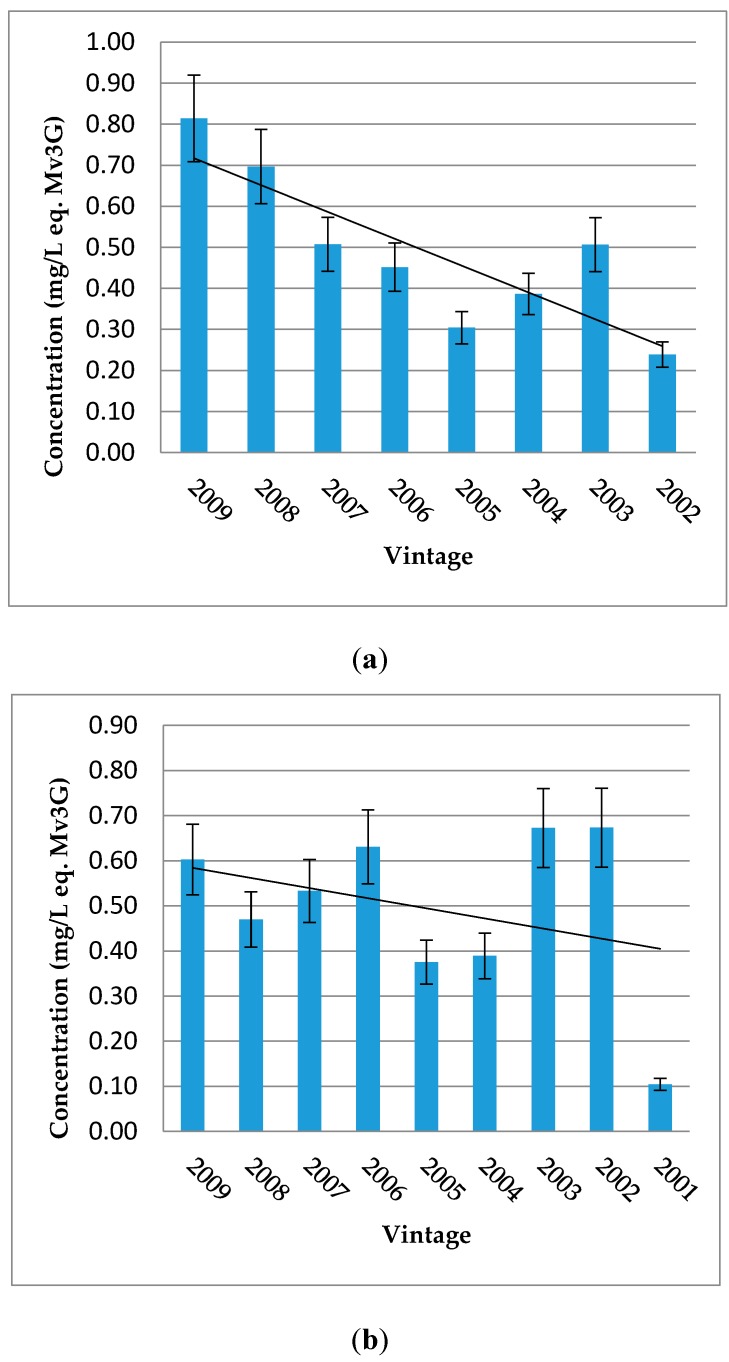
Distribution of vitisin A by vintage for each wine-growing region: Aquitaine (**a**) and Rioja (**b**). Error bars are standard deviation of wine samples within each category.

Médoc and Blayais wines are richer in vitisin A, catechin-Mv3G and pyranomalvidin-3-*O*-glucoside-4-vinylphenol derivatives. On the contrary, Rioja and “Bordeaux generic” samples are more concentrated in vitisin B, except for samples from Rioja Alta. Moreover, according to vintage for each wine-growing region, the tendency about the evolution of vitisin A shows a decrease in older wines (Figure 2).

#### 2.1.2. Tannin Analyses

Total tannin content (expressed in g/L) is presented in Table 1 and Table 2. Descriptive statistics are also evaluated for low molecular tannins (expressed in mg/L eq. (+)-catechin) (Table A2). (+)-Catechin contents are higher than (−)-epicatechin: they are twice higher in “Bordeaux specific” and “Bordeaux generic” samples, three times higher for Rioja Alta and Baja wines, and four times higher for Alavesa ones. Procyanidin dimer B1 is found in higher concentrations than procyanidin dimer B3 for each subregion.

“Bordeaux generic” and “Bordeaux specific” wines are richer in low molecular tannin contents than Rioja wines probably as a consequence of differences in varieties used in each region. The concentration in low molecular tannins ((+)-catechin, (−)-epicatechin, procyanidin dimers B1 and B3, and procyanidin trimer C1) is the highest in Graves samples (average concentration of 194.49 mg/L eq. (+)-catechin), whereas Alavesa wines show the lowest concentration (103.19 mg/L eq. (+)-catechin), that is twice less than in Graves samples. As regards Bordeaux wines, “Bordeaux generic” samples show the lowest concentrations (153.13 mg/L eq. (+)-catechin).

#### 2.1.3. Mean Degree of Polymerization

The mean degree of polymerization is commonly defined as the average number of base tannin units per molecule if the molecules were composed of regularly repeating units, or as the average number of monomeric tannin units per molecule. This value reaches 18 units in a grape seed fraction, and 30 units in grape skin extracts [26,27].

**Figure A2:**
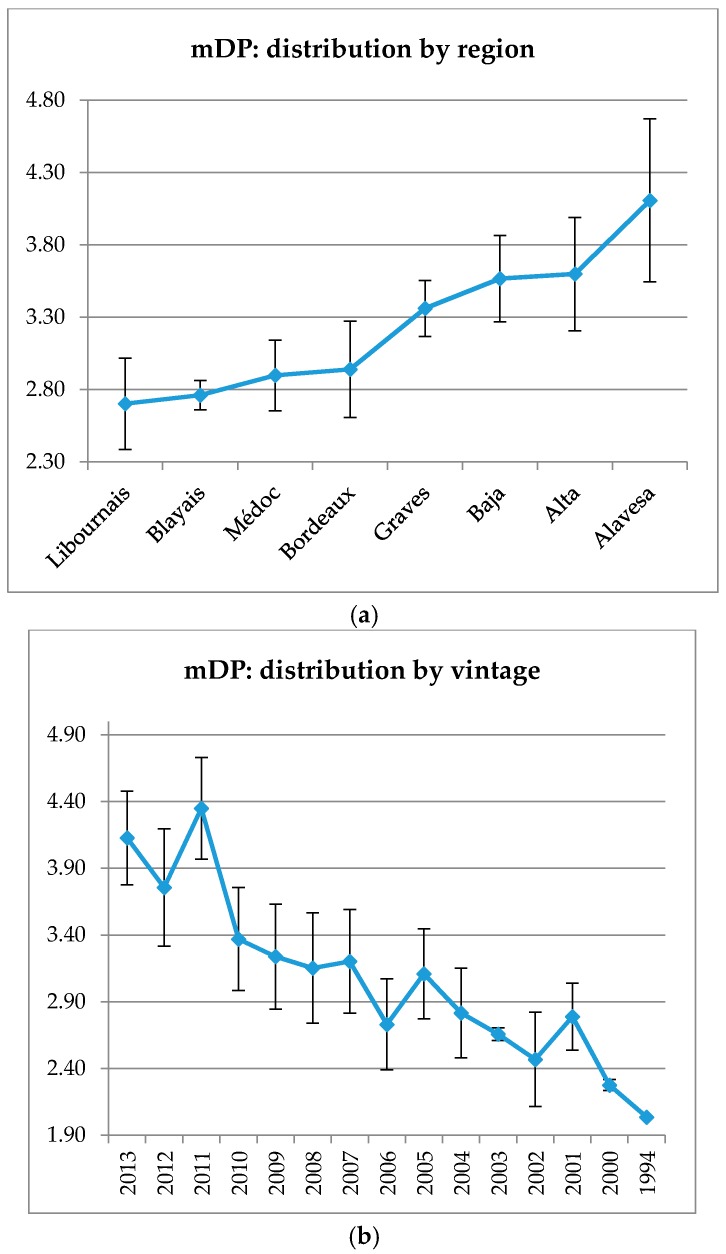
Mean degree of polymerization distributed by region (**a**) and vintage (**b**). Error bars are standard deviation of wine samples within each category.

**Figure A3:**
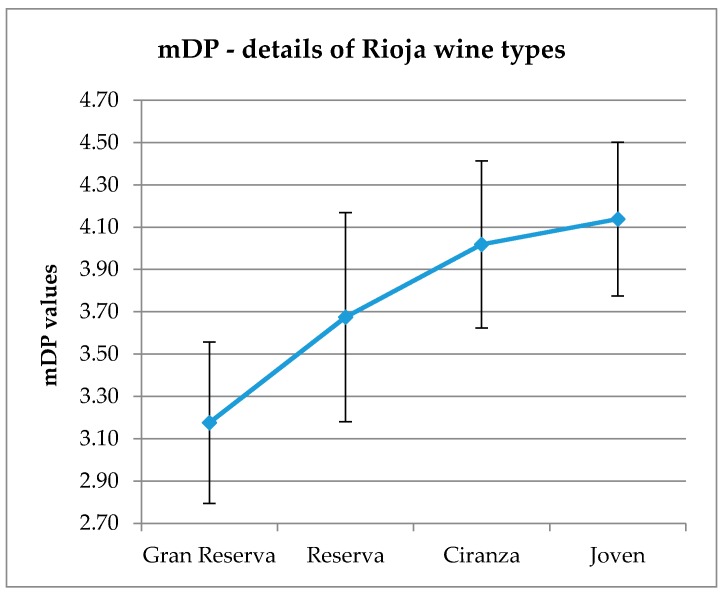
Mean degree of polymerization distributed by Rioja wine “types”. Error bars are standard deviation of wine samples within each category.

The mDP values for the different wine-growing regions vary from 2.7 to 4.16 (Figure A2). “Bordeaux generic” and “Bordeaux specific” subregions show the lowest mDP with a difference of 1 polymerization unit with Rioja wines (means of mDP 2.9 for all Bordeaux regions, mDP 3.8 for Rioja regions). Considering Rioja wines, mDP is well correlated with the wine “type” (Figure A3), a difference of 1 polymerization unit is observed between Gran Reserva and Joven wines (mDP 3.18 and 4.14, respectively). This tendency is also noticed from 2000 to 2013, with a decreasing mDP for older wines (Figure A2).

### 2.2. Statistical Significance

**Figure 3:**
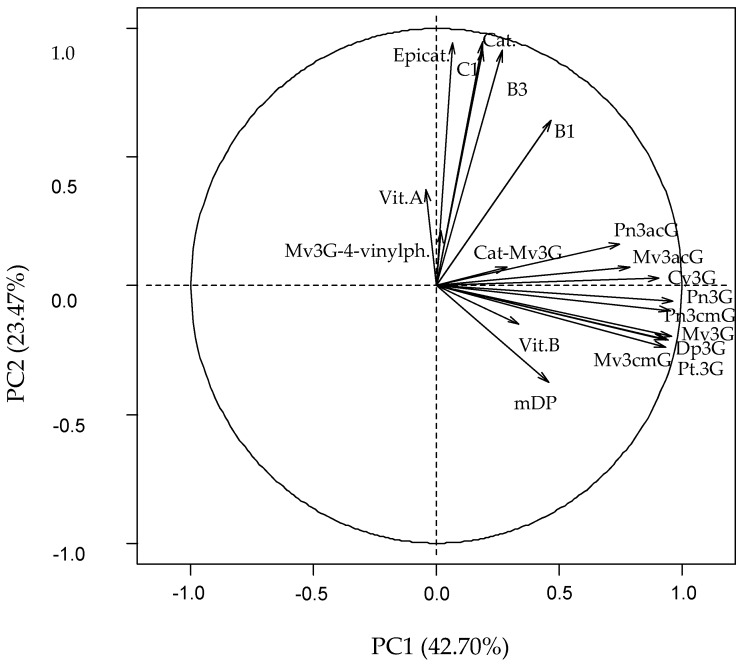
Principal component (PC) analysis. Distribution of samples along PC1 and PC2 and component weights of 19 variables. Mv3G-4-vinylph., pyranomalvidin-3-*O*-glucoside-4-vinylphenol; Vit.A, Vitisin A; Epicat., Epicatechin; C1, procyanidin trimer C1; Cat., Catechin; B3, procyanidin dimer B3; B1, procyanidin dimer B1; Cat-Mv3G, direct adduct catechin-malvidin-3-*O*-glucoside; Pn3acG, paeonidin-3-*O*-acetylglucoside; Mv3acG, malvidin-3-*O*-acetylglucoside; Cy3G, cyanidin-3-*O*-glucoside; Pn3G, paeonidin-3-*O*-glucoside; Pn3cmG, paeonidin-3-*O*-coumaroylglucoside; Mv3G, malvidin-3-*O*-glucoside; Dp3G, delphinidin-3-*O*-glucoside; Pt3G, petunidin-3-*O*-glucoside; Mv3cmG, malvidin-3-*O*-coumaroylglucoside; Vit.B, vitisin B; mDP, mean degree of polymerization.

**Figure 4:**
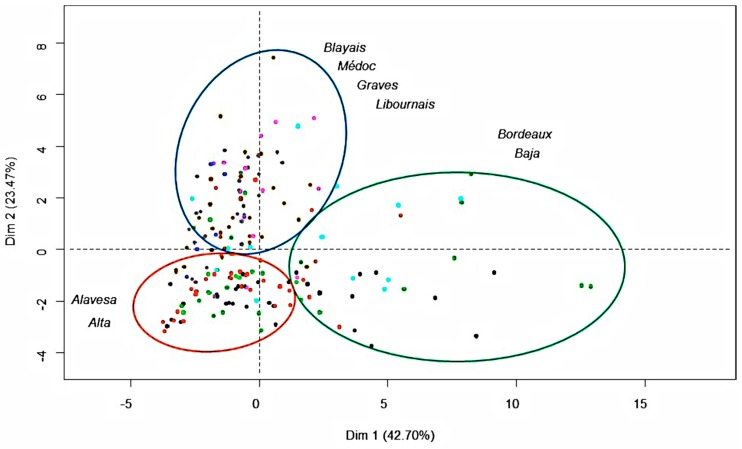
Distribution of the wine samples among PC1 (Dim 1) and PC2 (Dim 2).

**Figure A4:**
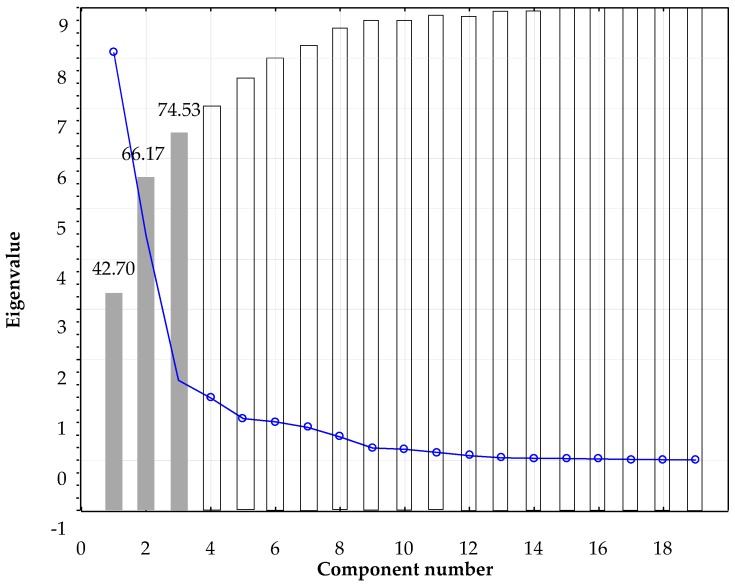
Principal components scree plot.

The results are confirmed when a principal component analysis over the whole data set was performed. From the nineteen components obtained, the first three with eigenvalues >1, were selected, accounting for almost 75% of the total variance (Figure A4). Figure 3 shows the component weights of 19 original variables, and Figure 4 shows the distribution of the 194 wine samples along the first two principal components (called PC1 and PC2 which explain 66.17% of the variance).

## 3. Discussion

### 3.1. Anthocyanin and Derived-Pigment Contents

The analysis of the anthocyanin composition and especially the acylation of anthocyanins can be used to discriminate different grape varieties, such as Tempranillo, Graciano and Cabernet Sauvignon [28,29]. Acetylated and *p*-coumaroylated pigments were considered to be important for the characterization of grape varieties [1,30,31]. In this study, for the Rioja region, red wines showed higher concentrations of *p*-coumaroyl derivatives but lower acetylated anthocyanin ones than those from “Bordeaux specific” subregions.

Nuñez and co-authors [29] showed significant differences between the anthocyanin distribution and acylation in grape skin extracts from Tempranillo, Graciano and Cabernet Sauvignon. The *p*-coumaroyl derivatives were found in higher quantities in Tempranillo, followed by Graciano and finally Cabernet Sauvignon, whereas acetylated derivatives were more abundant in Cabernet Sauvignon than the two others varieties [29]. Similar results have been also reported by Hebrero et al., who analyzed Tempranillo and compared the results with those obtained from Cabernet Sauvignon previously described by Wulf and Nagel [32,33].

Our results suggest that anthocyanin acylation can be used as a marker to distinguish wine samples in two groups in agreement with the grape variety used, regardless of the region of production. Most of the samples from Rioja, are made from Tempranillo and Graciano richer in *p-*coumaroyl derivatives, whereas Cabernet Sauvignon and Merlot are predominant in Bordeaux red wines, which explains the differences between both groups of wines.

Concerning pyranoanthocyanins and polymerized pigments, “Bordeaux specific” wines show a higher content in vitisin A than Rioja and “Bordeaux generic” wines, distributing the wine samples according to their origin and hence varieties, as initially shown for total and individual anthocyanin contents. The average content for the different vintages of Rioja Alta wines is smaller than that of the wine samples from the other areas (mean of each sample vintage, equal to 2007 which is the oldest mean), suggesting instability of vitisin B and a more stable behaviour of vitisin A, catechin-Mv3G and pyranoanthocyanin-4-vinylphenol adducts during ageing. Then, for Médoc and Graves regions, the proportion of Cabernet Sauvignon and the time of ageing in barrels are also more important than Libournais and Blayais (where Merlot is predominant). The evolution of the concentration in vitisin A in these samples show a decreasing trend for older wines, regardless of the wine-growing region (Figure 2). Such a decrease over ageing of vitisin A content was in agreement with the other results found in the literature [34,35,36,37,38]. The authors agreed that maximum of vitisin A content was reached shortly after fermentation, when pyruvic acid is still available, and was followed by a slow decline over ageing [30,31]. Moreover, in Port red wines, where vitisin A was the main pigment found during ageing [30,36], the decrease of this pigment was higher in the wines aged in oak barrels (15%–25%) than in the bottled samples (9%–18%). However, it is important to remain critical: for Aquitaine and Rioja wines, there was only one wine for the 2000 vintage. These wines were also not included in the figure since the concentrations in vitisin A were higher than in 2002 (Aquitaine) and 2001 (Rioja). The decreasing tendency found here will need to be confirmed in further studies.

### 3.2. Contents of Flavanol Monomer and Procyanidin Dimer and Trimer

In our wine samples the concentration in (+)-catechin is higher than in (−)-epicatechin, as previously described [39,40]. This trend is not typical for one grape variety in particular. Procyanidin dimer B1 is found in higher concentrations than procyanidin dimer B3 for all the wines. Dimer B1 is mainly found in grape skins, and also more extractable during wine-making than dimer B3 mainly located in grape seeds [41,42,43].

On the one hand, the lowest concentrations in catechin units, procyanidin dimers B1 and B3, and procyanidin trimer C1 were found in Alavesa wines. After studying the distribution of the grape varieties in Rioja regions, the majority of the red wines produced in Rioja Alavesa is composed of 100% Tempranillo whereas in the other Rioja regions wines were made with a blend of Tempranillo (70%–80%) and two other grape varieties: Graciano and Garnacha (10%–20% for each one). On the other side, low molecular tannin contents were found in the highest concentrations in Graves wines ((+)-catechin, (−)-epicatechin, procyanidin dimers B1 and B3, and procyanidin trimer C1). For Graves samples the predominant grape variety used for blend is Cabernet Sauvignon, in which the percentage of low molecular tannins was higher than in Merlot [41,44], which explains here that “Bordeaux generic” samples, that are mainly assembled with Merlot, show the lowest concentrations in low molecular tannins of all the Aquitaine wine-growing subregions.

Like the tendency observed for the anthocyanin analyses, the analysis of tannin composition also divides the samples into two groups: Rioja subregions and “Bordeaux generic” on the one hand and “Bordeaux specific” on the other hand, with a hierarchical richest decreasing approach according to the low molecular tannin contents in the grape varieties used for assembling: Cabernet Sauvignon, Merlot, Graciano and Garnacha, and Tempranillo.

### 3.3. Mean Degree of Polymerization

Using phloroglucinolysis, mDP values in the range of 1.5–2.5 and 1.3–7.1 have been reported for Bordeaux wines [45,46]. In the case of Bordeaux wines, the higher mDP value is found for Graves (3.36) whose wines are mainly made with Cabernet Sauvignon, which differentiates this region from the others. Previous studies investigated the discrimination of Cabernet Sauvignon from Merlot with the study of proanthocyanidin properties, especially by the way of the calculation of mDP values [47]. The highest contents in low molecular tannins (catechin units, dimers B1 and B3, and trimer C1), also found in Graves wines, can be correlated to the highest mDP values within “Bordeaux generic” and “specific” wines, explained by the predominance of Cabernet Sauvignon in such wines and the barrel ageing process.

A decrease of mDP values is observed for older wines: Gran Reserva samples show the lowest values, instead of Joven wines (the youngest samples). Chira [48] also reported this tendency with the highlight that mDP value was correlated to astringency, and that both parameters decreased over ageing, regardless of the grape variety.

However, the determination of mDP is based on a reaction in acidic medium, so ethylidene-bridged and oxidized structures may not be measured in this analysis. We presume that linkages from oxidized forms cannot be broken this way, and also that mDP values may differ from those enunciated.

### 3.4. Distribution of the Wines Samples by Principal Component Analysis

After principal component analysis (PCA), red wine samples are distributed in three groups according to two dimensions PC1 and PC2. The first dimension could be called “anthocyanin component”, because the variables characterizing the anthocyanin composition are grouped on the positive side of PC1. The variables in component 1 are the sum of the nine major monomers, the catechin-Mv3G adduct, vitisin B and mDP. They present positive weights and are strongly correlated to “Bordeaux generic” and Baja wine regions, most of whose wine samples are distributed in the positive part of this axis. The predominance of Merlot in blended “Bordeaux generic” wines, and the average age of wines from “Bordeaux generic” and Baja samples could explain why both regions are grouped together. Indeed, Merlot grape variety is in a greater proportion in wines than Cabernet Sauvignon and for this reason the samples are correlated to the anthocyanin component. Moreover, the average age of wines from both subregions is higher than for the other regions (2010 for Baja and 2011 for “Bordeaux generic”), and the proportion of monomeric anthocyanins is higher in younger wines.

The separation of the other wine regions is finally achieved with the help of PC2. The second dimension can be called “tannin component”, for the variables characterizing the tannin composition are grouped on the positive side of the PC2. The variables with a more important weight in component 2 are the monomers, dimers and trimer of flavan-3-ols. They present positive weights and are strongly correlated to Graves, Médoc, Libournais and Blayais regions, most of whose wine samples are located in the positive part of the axis. For Graves and Médoc, Cabernet Sauvignon is the main grape variety used for blending, which is rich in low molecular flavan-3-ols. The time of ageing in barrels could also support the grouping of these two subregions with Libournais and Blayais. However, information on the ageing conditions is not at our disposal.

Finally, most wine samples of the Rioja Alavesa and Alta regions are distributed on the opposite side, mainly due to their low relative tannin contents. The predominance of Tempranillo in the wines from these subregions also supports such a result. When observing ageing state within each group (from Gran Reserva to young wines in the case of Rioja and from older to more recent vintages for Aquitaine) a tendency to lower anthocyanins and anthocyanin-derivative pigments (lower PC1 values) and tannins (lower PC2 values) contents is clearly noticeable.

## 4. Materials and Methods

### 4.1. Reagents and Standards

Methyl alcohol and acetonitrile (Romil Chemical Ltd., Heidelberg, Germany), trifluoroacetic acid TFA (Merck; Darmstadt, Germany) and acetic acid AcOH (Merck) were HPLC grade. Concentrated aqueous hydrochloric acid (HCl) solution (32%) was from Merck. Deionised water was purified on a Milli-Q system (Millipore, Bedford, MA, USA). L(+)-Tartaric acid and ethyl alcohol of analytical grade were provided by Merck. NaH_2_PO_4_/Na_2_HPO_4_ 0.1 M solution buffer, which was prepared using monosodium phosphate (Sigma-Aldrich, Steinheim, Germany) and sodium phosphate dibasic (Merck), and sodium hydroxide NaOH (Panreac, Barcelona, Spain). Solution for phloroglucinolysis was prepared using phloroglucinol and l-ascorbic acid 99%+ reagent (Sigma-Aldrich, St. Louis, MO, USA), hydrochloric acid 37% and MeOH with HPLC grade (VWR, Fontenay-sous-Bois, France). Stop solution was prepared using sodium acetate (VWR) and Milli-Q water. Folin-Ciocalteau’s phenol reagent, sodium carbonate 99.5%+ granular and gallic acid monohydrate 98%+ were provided by Sigma-Aldrich. Standards of malvidin-3-*O*-glucoside (Mv-3-glc) and (+)-catechin were supplied by Extrasynthèse (Genay, France). All solvents used were previously filtered through 0.45 μm nylon membranes (Lida, Kenosha, WI, USA).

### 4.2. Wine Samples

A sampling of different red wines made at different times from both Aquitaine and Rioja was performed during January–June 2014 for this study: 94 Aquitaine red wines were from four subregions (wine-growing regions)—Médoc, Graves, Libournais and Blayais & Bourgeais, called “Bordeaux specific” in this study—and from two generic Bordeaux and Bordeaux-Supérieur appellations, called “Bordeaux generic”. 100 Rioja red wines were distributed in the three subregions: Rioja Alta, Rioja Baja and Rioja Alavesa. Rioja wines were divided depending on the time and way of ageing: 26 wines were qualified as young (Joven), 25 as Crianza (at least 12 months aged in oak barrels), 24 as Reserva (36 months of total ageing—barrels and bottles—with at least 12 months aged in oak barrels) and 25 as Gran Reserva (at least 24 months aged in oak barrels followed by 36 months of bottle ageing). The number of wine samples for each vintage and the average age for each subregion are presented in Table 3 and Table 4. All the wine samples were collected at the same time and all analyzed during summer 2014.

### 4.3. Anthocyanins and Pigment Contents

#### 4.3.1. Determination of Total Anthocyanins

Total anthocyanins are measured using bisulfite bleaching [49]. Two test tubes were filled with 10 mL of the following mixture: 1 mL of wine sample, 1 mL of EtOH acidified with 0.1% HCl and 20 mL of HCl concentrated at 2%. In the first tube 4 mL of H_2_O was added (DOref, optical density), and in the second 4 mL of sodium bisulfite at 15% (DOt). After 20 min of reaction, the optical density was measured at 520 nm for each tube, and the result was given following this relation: [C](mg/L) = 875 × (DO_t_ − DO_ref_).

#### 4.3.2. Analysis of Individual Anthocyanins

Individual anthocyanins were evaluated using the official method adopted by OIV (Vines and Wines International Organization) [50]. Determination of relative composition for the nine main individual anthocyanins in red wines was evaluated. Samples were directly filtered on a 0.45 µm membrane and analyzed using HPLC. The system used was an Accela series (Thermo-Scientific, Illkirch-Graffenstaden, France). The analysis was carried out on a 4 × 250 mm internal diameter (i.d.), 5 µm Nucleosil C18 column (Agilent, Les Ulis, France). The solvents used were A: H_2_O/HCOOH (95:5) and B: acetonitrile/HCOOH (95:5). The gradient consisted of: 10%–35% B in 25 min, and 35%–100% B in 1 min at a flow rate of 1 mL/min. The column was washed with 100% B for 5 min and re-equilibrated with the initial conditions for 5 min. Quantification was performed using an external standard of malvidin-3-*O*-glucoside (Mv3G).

#### 4.3.3. Analysis of Anthocyanin-Derived Pigments 

Wine samples were first clean up on Strata X cartridges (reversed-phase commercial Solid Phase Extraction (SPE) cartridges, Phenomenex, Torrance, CA, USA) previously conditioned with MeOH and synthetic wine, washed with 0.1 mol/L of phosphate buffer (pH = 6.5), which eluted phenolic acids, in order to reduce the effect of ionic saturation during HPLC-ESI-MS/MS analysis. The fraction of interest, eluted with acidic methanol (MeOH/HCl, 999:1), evaporated to dryness and solubilized in Milli-Q water acidified with TFA (995:5), was analysed with an Alliance 2695 HPLC instrument (Waters, Saint Quentin Yvelines, France) equipped with a 100 × 3.0 mm i.d., Onyx Monolithic C18 column (Phenomenex) thermostated at 30 °C. Detection was carried out at 530 nm, and complete spectral data were accumulated in the range 250–600 nm each second, using a Waters Alliance 2996 diode-array detector (DAD). Mass detection was carried out using a Quattro triple quadrupole mass spectrometer (Micromass, Guyancourt, France) equipped with a Z-spray electrospray ionization (ESI) source, performed in positive mode and without flow split, with the following parameters: capillary voltage 3.2 kV, extraction cone voltage 3 V, hexapole lenses voltage 0 V, source temperature 120 °C, desolvation temperature 300 °C and desolvation gas flow 450 L/h. The solvents used were solvent A: H_2_O/TFA (995:5 *v/v*) and B: CH_3_CN.The gradient consisted of 12% B isocratic during 0.29 min, linear gradient 12%–15% B in 4 min, then 15%–25% B from 4.29 to 9.17 min, 25%–40% B from 9.17 to 12.72 min, then isocratic 40% B from 12.72 to 13.17 min, finally 40%–100% B in 0.5 min. The column was washed with 100% B for 8 min and then reconditioned with initial conditions. The injection volume was 50 µL. The flow rate was 0.3 mL/min. Quantitation was performed using Multiple Reaction Monitoring (MRM) mode (Table 5). An external standard of Mv3G was used. [51].

### 4.4. Flavan-3-ol Contents

#### 4.4.1. Determination of Total Tannins

Total tannins were measured using the Bate-Smith reaction [52]. Two test tubes were filled with 2 mL of wine sample (dilution 1/50), 1 mL of distilled water and 3 mL of HCl 37%. One of the tubes was tightly capped and hydrolyzed at 100 °C during 30 min. After the hydrolysis, 500 µL of EtOH were added in each tube. The optical density (DO) was measured at 550 nm with an optical path length of 1 cm. Tannin concentration was given following this relation: [C](g/L) = 19.33 × ΔDO.

#### 4.4.2. Determination of Individual Tannins 

Tannins were analysed and measured with HPLC-ESI-MS(−)-CID-MS/MS (high-performance liquid chromatography-electrospray ionization collision-induced dissociation tandem mass spectrometry) analysis. Wine samples were previously fractionated on SPE system, which was a hydrophilic polymeric sorbent Oasis HLB (Waters Corporation). The obtained fractions of wine were solubilized in 1 mL of H_2_O/AcOH (99:1). HPLC material was described above (see Section 4.3.3). The mass parameters were: capillary voltage 2.6 kV in negative mode, source temperature 120 °C, extraction cone voltage 3 V, hexapole lenses voltage 0 V, source temperature 120 °C, desolvation temperature 300 °C and desolvation gas flow 450 L/h. The solvents used were solvent A: H_2_O/AcOH (99:1 *v/v*) and B: methyl alcohol/AcOH (99:1 *v/v*). The gradient consisted of 0% B during 1.03 min, from 0 to 20% B from 1.03 to 7.47 min, from 20% to 25% B from 7.47 to 11.25 min, from 25% to 45% B from 11.25 to 16.03 min, then from 45% to 75% B from 16.03 to 19.92 min, finally from 75% to 100% B from 19.92 to 24.70 min. The column was washed with 100% B during 3 min, then reconditioned with the initial conditions. The injection volume was 50 µL. The flow rate was 0.3 mL/min. [53]. Tannin quantification was performed using an external standard of (+)-catechin between the ranges of 0.005 and 150 mg/L, and MRM mode (Table 6).

### 4.5. Determination of Mean Degree of Polymerization

The proanthocyanidin mDP concentrations were quantified by phloroglucinolysis [45]. The oligomeric and polymeric proanthocyanidins were depolymerised in the presence of a nucleophilic agent phloroglucinol in an acidic medium. Reversed-phase HPLC analysis of the products formed allows determination of the structural composition of proanthocyanidins, which are characterised by the nature of their constitutive extension units (released as flavan-3-ols phloroglucinol adducts) and terminal units (released as flavan-3-ols). To calculate the apparent mDP, the sum of all subunits (flavan-3-ol monomer and phloroglucinol adducts, in mols) was divided by the sum of all flavan-3-ol monomers (in mols). Wine samples were analysed with a Surveyor series instrument (Thermo-Finnigan, Les Ullis, France) equipped with a 100 × 4.6 mm i.d., 3.5 µm X-Terra reversed-phase C18 column (Waters) thermostated at 25 °C. Detection was carried out at 280 nm using a Finnigan Surveyor PDA Plus detector. The mass detection was carried out using a Finnigan LCQ DECA XP MAX mass spectrometer with an ESI interface, performed in positive mode with the following parameters: capillary temperature 325 °C, capillary voltage 4 V, nebulizer gas flow 1.75 L/min, desolvation gas flow 1 L/min, and spray voltage 5 kV. The solvents used were solvent A: H_2_O/AcOH (99:1 *v/v*), and B: MeOH. The gradient consisted of 5% B during 25 min, linear gradient 5%–20% B in 20 min, then 20%–32% B in 15 min, finally 32%–100% B in 2 min. The column was washed with 100% B for 5 min and then stabilized with the initial conditions for 10 min. The injection volume was 20 µL. The flow rate was 1 mL/min.

### 4.6. Data Analysis

Statistical data analysis was performed using Statistica v.10 (Statsoft Inc., Tulsa, OK, USA). Descriptive statistics were realized for each chemical variable. Then a principal component analysis was performed using *R* to examine any possible grouping of samples according to wine-growing regions and vintages. PCA was performed on the correlation matrix using a few attributes that well characterize the phenolic composition of the wine samples.

## 5. Conclusions

This study shows significant differences between red wines from Rioja and those from Aquitaine. Although the samples are blended wines, there is a strong discrimination between the grape varieties, as previously described. *p*-coumaroylated pigments were found in higher concentrations in red wines from the Rioja area, for they are made from Tempranillo and Graciano varieties; whereas the proportion of acylated ones was higher in red wines from the Aquitaine area, in agreement with previous studies on the grape skin composition of Cabernet Sauvignon. Vitisin A content shows a decreasing trend when wines are older. The concentration of individual tannins is higher in “Bordeaux specific” red wines, especially for the samples where Cabernet Sauvignon is predominant.

According to the hierarchical ascendant classification, wine samples are scattered in three classes of wine regions and this result confirms the discrimination of wine regions according to PC1 and PC2. The first class effectively groups “Bordeaux generic” and Rioja Baja wines, which attributes were individual anthocyanins, mDP, polymerized pigments (catechin-Mv3G adduct) and vitisin B; the second class groups Blayais, Médoc, Graves and Libournais wines, characterized by monomers and dimers of flavan-3-ols, vinylphenol pyranoanthocyanins, and vitisin A; and finally, the last class is represented by Rioja Alavesa and Alta wines, contrary to the second class, since most of wine samples from these areas are mainly blended with Tempranillo. This separation by regions is mainly a consequence of the different varieties used by wine producers in each region. Ageing has a general effect of progressively decreasing anthocyanins, anthocyanin-derived pigment and tannins.

## Figures and Tables

**Table 1:** Total anthocyanin and tannin contents distributed by wine-growing region (**A**) and vintage (**B**).

**(A) Distribution by Wine-Growing Region**						
	**Total Anthocyanins (mg/L)**			**Total Tannins (g/L)**		
	**Mean**	**Std dev**	***N***	**Mean**	**Std dev**	***N***
**Blayais**	102.43	±51.09	5	4.37	±0.56	5
**Libournais**	135.71	±58.50	27	4.09	±1.15	27
**Médoc**	148.21	±52.93	36	4.42	±0.58	36
**Graves**	180.04	±51.15	13	4.05	±1.16	13
**Alta**	205.05	±92.75	37	4.13	±0.66	37
**Bordeaux**	207.31	±79.68	13	3.69	±0.89	13
**Alavesa**	227.39	±99.44	33	4.08	±0.73	33
**Baja**	249.86	±104.21	30	3.96	±0.84	30
**(B) Distribution by vintage**						
	**Total Anthocyanins (mg/L)**			**Total Tannins (g/L)**		
	**Mean**	**Std dev**	***N***	**Mean**	**Std dev**	***N***
2000	43.32	±31.65	2	3.50	±0.13	2
2001	254.02	±246.83	2	3.45	±0.23	2
2002	101.79	±68.09	2	3.52	±0.67	2
2003	96.90	±54.70	2	3.57	±0.11	2
2004	116.70	±69.21	8	4.61	±0.87	8
2005	156.18	±105.34	10	4.08	±0.73	10
2006	94.26	±35.41	8	4.89	±0.49	8
2007	156.19	±31.16	14	4.13	±0.40	14
2008	173.68	±85.96	19	4.45	±0.66	19
2009	175.19	±54.52	39	4.29	±0.91	39
2010	202.29	±55.48	49	4.10	±0.94	49
2011	276.16	±70.32	12	4.10	±0.52	12
2012	262.35	±72.31	14	3.75	±0.68	14
2013	332.51	±85.59	12	3.18	±0.41	12

Std dev, standard deviation; *N*, number of samples.

**Table 2:** Total anthocyanin and tannin contents distributed by subregion and vintages (**A**) Rioja subregion (**B**) Bordeaux subregion.

**(A) Distribution Vintages and Subregion for Rioja**						
**Subregion**	**Vintage**	**Total Anthocyanins (mg/L)**		**Total Tannins (g/L)**		***N***
		**Mean**	**Std dev**	**Mean**	**Std dev**	
**Alta**	2013	266.71	5.34	3.30	0.08	1
	2012	264.32	69.57	4.32	0.49	2
	2011	300.74	27.55	4.03	0.45	5
	2010	217.37	63.66	3.95	0.34	9
	2009	229.42	42.27	5.23	0.29	3
	2008	159.12	71.09	4.78	0.65	2
	2007	169.01	58.43	4.04	0.42	5
	2006	111.85	3.50	5.33	0.18	1
	2005	134.41	55.53	4.55	0.66	3
	2004	75.95	54.13	3.58	0.14	2
	2001	40.29	0.68	3.26	0.11	1
**Alavesa**	2013	324.14	84.91	3.49	0.14	3
	2012	312.94	56.11	3.40	0.76	5
	2011	356.08	98.21	4.75	0.27	2
	2010	251.59	75.80	3.99	0.60	6
	2009	198.58	36.58	4.72	0.49	4
	2008	135.07	54.22	3.82	0.26	2
	2007	213.47	53.94	4.30	0.40	3
	2006	147.91	4.63	5.21	0.18	1
	2005	102.40	44.76	3.95	0.86	3
	2004	171.52	70.30	4.57	0.70	3
	2000	70.66	4.02	3.58	0.12	1
**Baja**	2013	361.05	82.34	3.01	0.46	7
	2011	219.62	40.02	3.91	0.50	5
	2010	176.63	41.97	4.41	0.23	3
	2009	207.93	64.32	4.43	0.83	4
	2008	299.42	65.81	5.11	0.64	4
	2007	114.63	62.07	3.91	0.34	4
	2006	143.62	4.50	4.09	0.14	1
	2005	294.07	143.88	3.37	0.48	2
	2003	144.16	4.52	3.62	0.12	1
	2002	160.71	5.03	2.96	0.10	1
	2001	67.75	7.71	3.64	0.12	1
**(B) Distribution by Vintages and Subregion for Bordeaux**						
**Subregion**	**Vintage**	**Total Anthocyanins (mg/L)**		**Total Tannins (g/L)**		***N***
		**Mean**	**Std dev**	**Mean**	**Std dev**	
**Blayais**	2009	160.41	10.01	4.93	0.31	2
	2008	68.17	13.51	3.81	0.16	2
	2005	54.12	2.42	4.39	0.15	1
**Libournais**	2010	181.81	37.56	4.27	1.19	11
	2009	128.54	33.34	3.84	1.26	10
	2008	108.71	35.36	4.23	0.48	2
	2004	69.83	0.87	5.87	0.07	1
	2003	49.66	5.14	3.53	0.12	1
	2000	15.99	0.70	3.42	0.12	1
**Médoc**	2010	197.38	33.47	4.61	0.52	8
	2009	167.08	34.14	4.24	0.69	14
	2008	127.23	29.19	4.35	0.52	5
	2007	114.60	6.48	4.55	0.18	2
	2006	68.26	14.31	4.60	0.28	3
	2005	107.09	4.71	4.19	0.15	1
	2004	98.69	49.27	5.08	0.43	2
	2002	42.88	0.62	4.08	0.30	1
**Graves**	2010	199.69	29.56	3.78	1.35	8
	2009	240.84	9.47	4.11	0.11	1
	2008	156.47	29.91	4.56	0.65	2
	2007	128.15	5.64	3.91	0.14	1
	2006	61.12	5.38	5.26	0.02	1
**“Bordeaux generic”**	2013	223.60	9.84	3.37	0.11	1
	2012	224.03	69.84	3.76	0.55	6
	2010	185.01	83.56	3.29	1.15	4
	2009	302.39	11.89	3.50	0.08	1
	2006	84.83	5.51	5.39	0.11	1

Std dev, standard deviation; *N*, number of samples.

**Table 3:** Rioja wines by subregion and style.

Subregion	Average Age	Range Age	*N*	Style	Average Age	Range Age	*N*
**Alta**	2008	2001–2013	34	**Young**	2011	2007–2013	9
				**Crianza**	2010	2010–2011	9
				**Reserva**	2007	2004–2009	8
				**Gran Reserva**	2005	2001–2008	8
**Alavesa**	2009	2000–2013	33	**Young**	2012	2012–2013	8
				**Crianza**	2010	2009–2011	8
				**Reserva**	2008	2005–2010	8
				**Gran Reserva**	2005	2000–2007	9
**Baja**	2009	2001–2013	33	**Young**	2013	2011–2013	9
				**Crianza**	2010	2009–2011	8
				**Reserva**	2008	2007–2009	8
				**Gran Reserva**	2005	2001–2008	8

*N*, number of samples.

**Table 4:** Bordeaux wines by subregion.

Subregion	Average Age	Range Age	*N*
**Blayais**	2008	2005–2009	5
**Libournais**	2008	2000–2010	26
**Médoc**	2008	2002–2011	36
**Graves**	2009	2006–2010	13
**“Bordeaux generic”**	2011	2006–2013	13

*N*, number of samples.

**Table 5:** Analysis of anthocyanin-derived pigments: parameters for multiple reaction monitoring (MRM).

Analyte	Mass Transition	Cone Voltage (V)	Collision Energy (eV)
**Mv3G-acetaldehyde**	517→355	25	25
**Mv3*p*-coumG-acetaldehyde**	663→355	35	25
**Mv3G-pyruvic acid**	561→399	25	25
**Mv3*p*-coumG-pyruvic acid**	707→399	35	25
**Mv3G-vinylmethyl**	531→369	25	25
**Mv3G-vinylphenol**	609→447	25	25
**Mv3*p*-coumG-vinylphenol**	755→447	35	25
**Mv3G-vinylcatechol**	625→463	35	25
**Mv3G-vinylguaiacol**	639→447	35	25
**Mv3*p*-coumG-vinylguaiacol**	785→447	35	35
**Cat-Mv3G**	781→619	35	25
**Cat-Mv3*p*-coumG**	927→619	35	25
**Epicat-Mv3G**	781→619	35	25
**(Epi)Gallocat-Mv3G**	797→635	35	25
**Mv3*p*-coumG-8-ethyl-(epi)cat**	955→665	35	25

**Table 6:** Determination of individual tannins: parameters of multiple reaction monitoring.

Molecule	Mass Transition	Cone Voltage (V)	Collision Energy (eV)
(+)-catechin	289→137	25	25
(−)-epicatechin	289→137	25	25
(+)-gallocatechin	305→137	25	25
(−)-epigallocatechin	305→137	25	25
Procyanidin B dimers	577→289	25	25
Prodelphinidin B dimers	609→305	25	25
Mixed B dimers	593→305	35	25
Procyanidins B trimers	865→577	25	25
Procyanidin A dimers	575→285	15	25
Mixed A dimers	591→303	55	25
Mixed trimers with one A bond and one B bond	879→591	35	25
Vinyl-flavan-3-ols	315→163	55	25
Procyanidin dimers with furfuryl bridge	657→369	55	25
Glycosylated flavan-3-ols	451→289	55	25

## Appendix A

**Table A1:** Descriptive statistics of the anthocyanin and derived pigment contents for each wine region, expressed in mg/L in equivalent malvidin-3-*O*-glucoside.

*N*	Alavesa 33		Alta 34		Baja 33		Blayais 5		Bordeaux 13		Graves 13		Libournais 27		Médoc 36	
Dp3G	6.53	±5.70	4.43	±3.55	7.58	±7.10	0.95	±0.34	5.59	±3.99	3.15	±1.77	1.94	±1.32	2.21	±1.41
Cy3G	0.83	±0.59	0.67	±0.28	1.53	±3.25	0.47	±0.09	1.00	±0.52	0.71	±0.18	0.63	±0.23	0.67	±0.25
Pt3G	6.20	±5.28	4.21	±3.63	7.41	±7.27	0.75	±0.24	4.56	±2.98	2.64	±1.77	1.70	±1.58	1.80	±1.11
Pn3G	2.75	±2.34	2.00	±1.42	4.51	±4.65	0.96	±0.18	3.63	±2.32	1.82	±0.84	1.26	±0.66	1.44	±0.93
Mv3G	23.42	±20.7	15.27	±14.43	28.44	±26.93	2.32	±1.11	21.34	±13.57	11.94	±8.88	5.48	±3.69	8.00	±4.88
Pn3acG	0.55	±0.23	0.47	±0.17	1.21	±3.25	0.41	±0.05	1.32	±0.69	0.68	±0.23	0.52	±0.19	0.57	±0.18
Mv3acG	2.43	±1.85	1.81	±1.41	3.13	±3.40	1.04	±0.34	6.58	±4.08	2.56	±1.47	1.61	±1.01	2.22	±1.39
Pn3cmG	0.69	±0.28	0.58	±0.22	1.42	±3.23	0.45	±0.08	0.89	±0.34	0.60	±0.10	0.49	±0.10	0.51	±0.11
Mv3cmG	2.68	±1.82	1.99	±1.67	3.76	±3.69	0.75	±0.25	2.54	±1.40	1.51	±1.00	0.99	±0.42	1.21	±0.59
Vit. A	0.56	±0.17	0.43	±0.22	0.46	±0.20	0.88	±0.25	0.53	±0.19	0.65	±0.21	0.76	±0.35	0.81	±0.35
Vit. B	0.10	±0.09	0.05	±0.04	0.15	±0.15	0.04	±0.02	0.13	±0.17	0.07	±0.06	0.06	±0.05	0.06	±0.05
Mv3G-4-vinylph.	0.43	±0.05	0.41	±0.04	0.43	±0.05	0.46	±0.04	0.52	±0.05	0.59	±0.07	0.46	±0.07	0.55	±0.06

*N*, Number of samples; Dp3G*,* delphinidin-3-*O*-glucoside; Cy3G, cyanidin-3-*O*-glucoside; Pt3G, petunidin-3-*O*-glucoside; Pn3G, paeonidin-3-*O*-glucoside; Mv3G, malvidin-3-*O*-glucoside; Pn3acG, paeonidin-3-*O*-acetylglucoside; Mv3acG, malvidin-3-*O*-acetylglucoside; Pn3cmG, paeonidin-3-*O*-coumaroylglucoside; Mv3cmG, malvidin-3-*O*-coumaroylglucoside; Vit.A, vitisin A; Vit.B, vitisin B; Mv3G-4-vinylph, pyranomalvidin-3-*O*-glucoside-4-vinylphenol.

**Table A2:** Descriptive statistics of the tannin content for each wine region, expressed in mg/L in equivalent (+)-catechin.

*N*	Alavesa 33		Alta 34		Baja 33		Blayais 5		Bordeaux 13		Graves 13		Libournais 27		Médoc 36	
Cat-Mv3G	0.09	±0.04	0.06	±0.04	0.07	±0.04	0.05	±0.03	0.07	±0.02	0.10	±0.04	0.08	±0.03	0.10	±0.04
(+)-cat.	42.97	±9.47	49.48	±16.86	54.75	±18.39	75.32	±21.76	64.93	±18.91	77.37	±17.57	69.92	±15.67	66.29	±13.90
(−)-epicat.	9.78	±4.94	14.82	±11.80	19.48	±10.74	31.67	±15.74	29.70	±17.01	38.25	±14.54	32.95	±10.58	30.63	±12.55
B1	30.65	±9.12	30.24	±12.02	29.05	±11.88	30.36	±8.79	30.68	±9.18	40.77	±9.80	33.15	±10.99	31.04	±10.89
B3	10.37	±3.77	12.17	±6.69	14.01	±7.19	15.17	±7.47	17.85	±9.14	25.63	±10.71	21.08	±8.30	19.17	±9.26
C1	5.17	±0.90	5.59	±1.60	6.08	±1.90	7.01	±1.54	6.96	±1.82	9.01	±2.22	7.83	±1.84	7.39	±1.88
mDP	4.16	±1.15	3.62	±0.82	3.50	±0.61	2.76	±0.22	2.94	±0.68	3.36	±0.40	2.70	±0.64	2.90	±0.49

*N*, Number of samples; Cat-Mv3G, catechin-malvidin-3-*O*-glucoside; (+)-cat., (+)-catechin; (−)-epicat*.,* (*−*)-epicatechin; B1, procyanidin dimer B1; B3, procyanidin dimer B3; C1, procyanidin trimer C1; mDP, mean degree of polymerisation.
